# Reliability assessment of the upper urinary tract dilation grading system based on magnetic resonance urography in patients with neurogenic bladder

**DOI:** 10.1186/s12894-022-01039-y

**Published:** 2022-07-04

**Authors:** Zhonghan Zhou, Limin Liao, Xing Li, Xuesheng Wang, Xunhua Li, Hui Zhao, Han Deng, Qinggang Liu, Yi Gao, Huafang Jing

**Affiliations:** 1 Cheeloo College of Medicine, Shandong University; Department of Urology, China Rehabilitation Research Center, Beijing, 100068 China; 2 University of Health and Rehabilitation Sciences, Qingdao, Shandong China; 3 School of Rehabilitation, Capital Medical University, Beijing, China

**Keywords:** Upper urinary tract dilation, Magnetic resonance urography, Reliability, Neurogenic bladder

## Abstract

**Background:**

To assess the inter-observer and intra-observer reliability of the magnetic resonance urography (MRU)—upper urinary tract dilation (UUTD) grading system.

**Methods:**

A total of 40 patients with a diagnosis of NB were enrolled in this study. The images were assembled in an electronic presentation randomly. The presentations were reviewed and graded by 4 junior and 4 senior urologists. One week later, the images were randomized again and reassessed. The inter-observer reliability was estimated by Kendall’s coefficient of concordance and intra-class correlation coefficient (ICC), and the intra-observer reliability was estimated by weighted Cohen’s kappa.

**Results:**

The inter-observer reliability strength was excellent for all urologists, with the ICC value of 0.939 (0.908–0.963) and Kendall’s W value of 0.967. The highest agreement was shown in Grade 4 at 92.50%, and the lowest in Grade 2 at 82.14%. All disagreements were within one grade of difference. Moreover, the Intra-observer reliability was excellent, with the weighted kappa value ranging from 0.904 to 0.954.

**Conclusions:**

The inter-observer and intra-observer reliability of this novel MRU-UUTD grading system is confirmed, providing adequate evidence for broader clinical application.

## Background

Neurogenic bladder (NB) is an intractable urinary disorder with abnormal storage and micturition caused by various nervous system disorders [1]. Incontinence, hydronephrosis (HN), ureteral dilation (UD), and vesicoureteral reflux (VUR) are several complications caused by high bladder pressure in NB patients. Upper urinary tract dilation (UUTD), which refers to HN and UD, generates a burden on renal function and might lead to chronic renal failure [2]. An accurate evaluation of the degree of UUTD is important because further clinical decisions are dependent on the severity of UUTD. In 1993, the Society for Fetal Urology (SFU) established a classification system based on ultrasonography [3]; however, the SFU system has not been widely applied among clinical urologists. The SFU system also demonstrated deficiencies that are especially evident in the differentiation of severe hydronephrosis [grades 3 and 4] [4]. In 2014 Liao [5] described a novel UUTD grading system based on magnetic resonance urography (MRU). This new system more precisely discriminates among grade changes in upper urinary tract function, allowing for better informed clinical decision-making and long-term follow-up. Despite the merits of the novel UUTD grading system, the inter- and intra-observer reliability of this tool has not been investigated. A grading system cannot be considered valid without sufficient reliability. Therefore, we conducted this analysis to assess the inter- and intra-observer reliability of the MRU-UUTD grading system and provided adequate evidence for broader clinical application.

## Methods

The current study was a retrospective observational study involving 40 patients (24 males and 16 females; mean age: 25 years, range: 13–65 years) who presented with a diagnosis of NB at the China Rehabilitation Research Center. The most common etiologies were neural tube defect (19 patients), followed by intraspinal tumor (6 patients), lumbar disc herniation (4 patients), traumatic spinal cord injury (4 patients), and others (7 patients). The mean duration of their lower tract symptoms was 13.1 years (range: 3–40 years). The study was approved by the Institute Board. The images were assembled in a Microsoft Office Word presentation in random order. All identifying information was removed to reduce possible bias. The UUTD was classified into five grades based on coronal and transverse images, and the maximum intensity projection on MRU (Table 1). The presentations were reviewed and graded by 4 senior (with > 5 years of experience in urology, labeled as reviewer A-D) and 4 junior (with ≤ 5 years of experience in urology, labeled as reviewer E–H) urologists. One week later, the images were randomized again and reassessed by three urologists (reviewer F–H). All grading was performed independently.

**Table 1:** Comparison of the MRU-UUTD grading system with the SFU grading system

	MRU-UUTD grading system	SFU grading system
Grade 0	① Closely apposed central renal complex; ② No ureteral dilation	No hydronephrosis
Grade 1	① Slightly separation of the central renal complex without visualized calices ② Dilated Ureter, < 7 mm in diameter	Visible renal pelvis without calices
Grade 2	① Further dilated renal pelvis, a single or a few visualized calices; ② Dilated Ureter, < 10 mm in diameter	Hydronephrosis with a single or a few visualized calices
Grade 3	① Fully dilated renal pelvis, fluid-filled calices throughout the kidney; ② Thinned renal parenchyma overlying the calices (< 50%) ③ Tortuous Ureter, < 15 mm in diameter	Hydronephrosis with fluid-filled calices throughout the kidney
Grade 4	① Fully dilated renal pelvis, fluid-filled calices throughout the kidney; ② Thinned renal parenchyma overlying the calices (> 50%) ③ Tortuous Ureter, > 15 mm in diameter	Hydronephrosis with fluid-filled calices throughout the kidney, together with thinned renal parenchyma

Statistical analysis was performed using R (version 4.1.1; The R Foundation, Vienna, Austria). The inter-observer reliability of the MRU-UUTD grading system was estimated by Kendall’s coefficient of concordance and the intraclass correlation coefficient (ICC), and the intra-observer reliability was estimated by the weighted Cohen’s kappa. A *P* value < 0.05 was considered statistically significant.

## Results

### Inter-observer reliability of the MRU-UUTD grading system

**Fig. 1:**
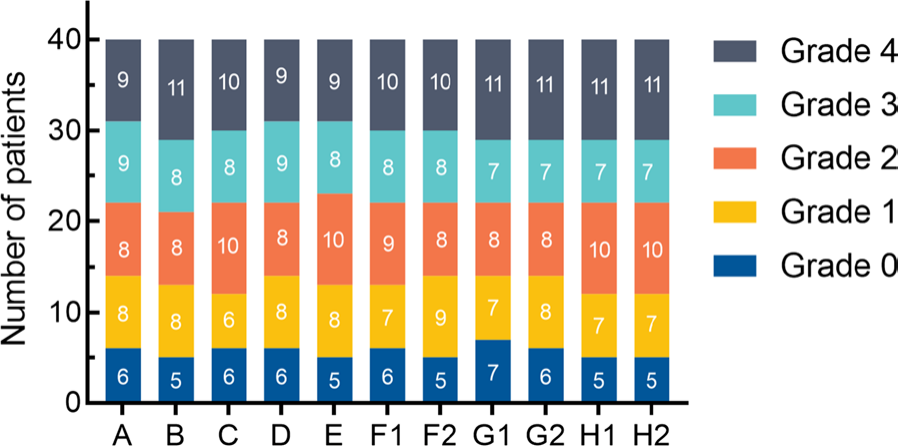
The number of MRU-UUTD grades assessed by each reviewer

**Fig. 2:**
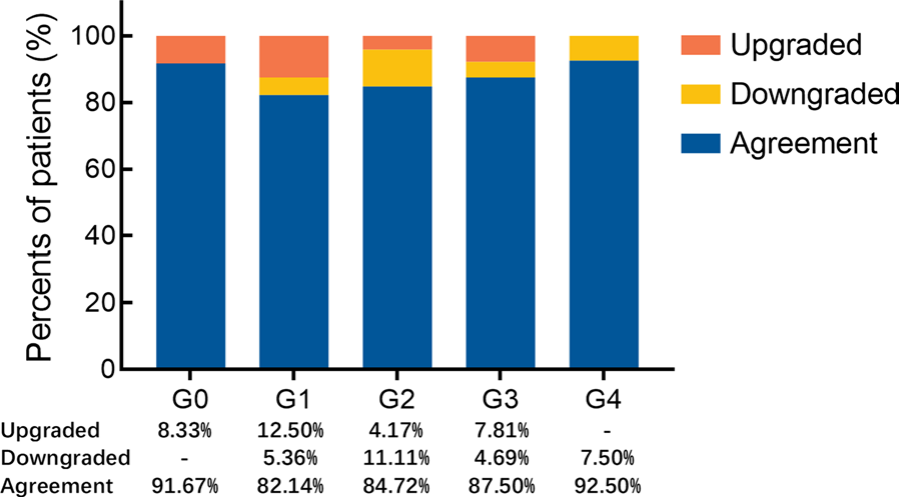
Inter-observer agreement for each grade

A total of 440 observations were performed. The number of MRU-UUTD grades assessed by each reviewer is summarized in Fig. 1. The agreement strength was excellent for all urologists, with an ICC value of 0.939 (0.908–0.963) and Kendall’s W value of 0.967 (Table 2). Subgroup analysis showed that both junior and senior urologists had excellent discrimination for different grades of UUTD, and senior urologists performed slightly better than junior urologists. The highest and lowest agreement was shown for grade 4 (92.50%) and grade 2 (82.14%; Fig. 2), respectively. All disagreements were within one grade of difference, with a range of 7.50%-17.86%.

**Table 2:** Inter-observer reliability values

	ICC	95%CI	*P*	Kendall's W	*P*	Agreement strength
All	0.939	0.908–0.963	< 0.001	0.967	< 0.001	Excellent
Seniors	0.957	0.932–0.975	< 0.001	0.948	< 0.001	Excellent
Juniors	0.914	0.866–0.949	< 0.001	0.939	< 0.001	Excellent

### Intra-observer reliability of the MRU-UUTD grading system

**Fig. 3:**
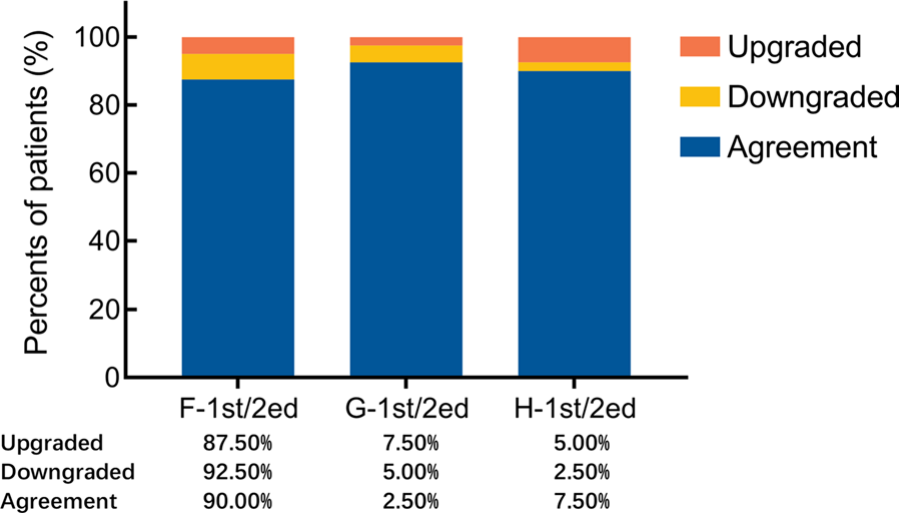
Intra-observer agreement for each reviewer

The intra-observer reliability was assessed by three urologists. The agreements were excellent for all three urologists, with the weighted kappa value ranging from 0.904 to 0.954 (Table 3). A one-grade difference occurred in 7.50–12.50% assessments (Fig. 3).

**Table 3:** Intra-observer reliability values

	Weighted Kappa	*P*	Agreement strength
Reviewer F-1st/2ed	0.904	< 0.001	Excellent
Reviewer G-1st/2ed	0.954	< 0.001	Excellent
Reviewer H-1st/2ed	0.935	< 0.001	Excellent

## Discussion

Ultrasonography is the basic and most commonly used method for classifying the degree of HN 3. The SFU grading system has been widely used; however, the SFU grading system has several deficiencies. A previous study has reported that the SFU grading system had a perfect intra-observer agreement, but modest inter-observer agreement [6]. This finding might be caused by the wide range of grade 4 definitions. Renal parenchyma loss is a long-term chronic course, thus parenchyma thinning might occur in some patients with SFU grade 2 or 3. Other attempts have been made to improve the SFU system based on ultrasonography [7–9]. For example, Onen [7] introduced a new grading system based on ultrasonography in 2007. Onen [7] divided the SFU grade 4 into Onen grades 3 and 4 according to the criterion of 50% parenchyma loss; however, none of these improvements have been widely accepted. Moreover, because ultrasonography is usually performed by sonographers, the images are not easy for clinical urologists to interpret and the description for UUTD is somewhat subjective. In 2014, Liao [5] described a novel MRU-UUTD grading system that integrated the severe forms of HN and UD. Like Onen, we modified the definition of SFU grades 3 and 4 by adding the parameter of moderate renal parenchyma thinning to grade 3. We thus emphasized the long process of parenchyma thinning, and thus better discriminated among higher grade changes in upper urinary tract function. Such grading can be applied for surveillance of UUTD, including both pre- and post-operative observations [10]. Moreover, the novel system is based on the coronal and transverse image panel and the maximum intensity projection in MRU. MRU is easier and more understandable, and it provides much more information for urologists about the disease than ultrasonography.

In our previous study, we correlated the MRU-UUTD system with the SFU system and showed that there was no significant difference between the two systems, but a significant difference was detected between grades 3 and 4 [5]; however, there has been no investigation into the reliability, which is essential for broader clinical application. In the present study, we confirmed the inter- and intra-observer agreement of the MRU-UUTD grading system. Excellent performances were observed among both senior and junior urologists. Intra-observer reliability was evaluated in 3 urologists 7 days after the first assessment, and substantial agreement was detected as well. All disagreements were within one grade of difference in both inter- and intra-observer assessments, suggesting the system is stable and discriminative.

In the subgroup analysis, we found that the agreement between grades 1 and 2 was lower than grades 3 and 4. A previous study [6] has reported more variability in discriminating HN with only renal pelvis dilatation (SFU grade 1) and HN with a single or a few visualized calices (SFU grade 2). In the present study, we showed that 12.5% of grade 1 cases were mistakenly upgraded to grade 2, while 11.1% of grade 2 cases were mistakenly downgraded to grade 1, which is not consistent with the SFU grading system. Using the Onen system, which also added the criterion of 50% parenchyma loss, it was reported that inter-observer agreement was lower for Onen grade 2 [all calyceal dilation] and grade 3 [all calyceal dilation plus < 50% parenchymal loss] [4]; however, we did not discriminate in the MRU-UUTD grading system whether a mild parenchymal loss was present or not, which has no clear definition for the onset of the chronic process. Therefore, the agreement rate for grades 2 and 3 was relatively high.

However, there are also limitations in our study. Firstly, this is a retrospective study, and the reviewers were aware of the purpose of the research, therefore, reviewer’s bias might occur. Secondly, the sample size (*n* = 40) is small, and more research is required for further evaluation. However, we performed the sample size calculation with PASS software. A sample size of 19 subjects with 8 observations per subject achieved 91% power to detect an intraclass correlation of 0.900. In our research, we enrolled 40 patients, and the power would be strong enough for a reliable conclusion. Thirdly, the etiology spectrum of NB is wide, and the nature, extent, and duration of the disease may influence the manifestation of UUTD. The patients enrolled in this study were not consecutive, because patients with severe UUTD accounted for a larger proportion compared with the mild to moderate UUTD in our institution. However, in this research, we intended to provide a series of patients of different grades with equal proportions for reliability assessment. We excluded some patients, which might lead to the unrepresentative of the sample. Therefore, the reliability of the system in a different population should be confirmed by further studies.

## Conclusions

We conducted a retrospective observational study to assess the reliability of the MRU-UUTD grading system in NB patients. The inter- and intra-observer reliability of this novel system was confirmed, offering more evidence for broader application. The discrimination power and effectiveness for longitudinal monitoring of UUTD warrants verification in corollary studies.

## Data Availability

The datasets generated and/or analyzed during the current study are not publicly available due protecting participant confidentiality but are available from the corresponding author on reasonable request.

## Abbreviations

NB: Neurogenic bladder
HN: Hydronephrosis
UD: Ureteral dilation
VUR: Vesicoureteral reflux
UUTD: Upper urinary tract dilation
SFU: The society for fetal urology
MRU: Magnetic resonance urography
ICC: Intraclass correlation coefficient

## References

[1] Liao L (2015). Evaluation and management of neurogenic bladder: what is new in China?. Int J Mol Sci.

[2] Farrugia MK, Whitaker RH (2019). The search for the definition, etiology, and effective diagnosis of upper urinary tract obstruction: the Whitaker test then and now. J Pediatr Urol.

[3] Fernbach SK, Maizels M, Conway JJ (1993). Ultrasound grading of hydronephrosis: introduction to the system used by the society for fetal urology. Pediatr Radiol.

[4] Kim SY, Kim MJ, Yoon CS, Lee MS, Han KH, Lee MJ (2013). Comparison of the reliability of two hydronephrosis grading systems: the Society for Foetal Urology grading system vs. the Onen grading system. Clin Radiol.

[5] Liao L, Zhang F, Chen G (2014). New grading system for upper urinary tract dilation using magnetic resonance urography in patients with neurogenic bladder. BMC Urol.

[6] Keays MA, Guerra LA, Mihill J (2008). Reliability assessment of Society for fetal urology ultrasound grading system for hydronephrosis. J Urol.

[7] Onen A (2007). An alternative grading system to refine the criteria for severity of hydronephrosis and optimal treatment guidelines in neonates with primary UPJ-type hydronephrosis. J Pediatr Urol.

[8] Venkatesan K, Green J, Shapiro SR, Steinhardt GF (2009). Correlation of hydronephrosis index to society of fetal urology hydronephrosis scale. Adv Urol.

[9] Rodriguez LV, Lock J, Kennedy WA, Shortliffe LM (2001). Evaluation of sonographic renal parenchymal area in the management of hydronephrosis. J Urol.

[10] Liao L, Zhang F, Chen G (2014). Midterm outcomes of protection for upper urinary tract function by augmentation enterocystoplasty in patients with neurogenic bladder. Int Urol Nephrol.

